# Generating roots of cubic polynomials by Cardano's approach on correspondence analysis

**DOI:** 10.1016/j.heliyon.2020.e03998

**Published:** 2020-06-11

**Authors:** Karunia E. Lestari, Udjianna S. Pasaribu, Sapto W. Indratno, Hanni Garminia

**Affiliations:** a Faculty of Mathematics and Natural Sciences, Institut Teknologi Bandung, Jalan Ganesha 10, Bandung 40132, Indonesia; b Statistics Research Division, Faculty of Mathematics and Natural Sciences, Institut Teknologi Bandung, Jalan Ganesha 10, Bandung 40132, Indonesia; c Algebra Research Division, Faculty of Mathematics and Natural Sciences, Institut Teknologi Bandung, Jalan Ganesha 10, Bandung 40132, Indonesia

**Keywords:** Mathematics, Cardano's formula, Contingency table, Correspondence analysis, Singular value decomposition

## Abstract

Cardano's formula is among the most popular cubic formula to solve any third-degree polynomial equation. In this paper, we propose the Cardano's approach as the alternative solution to generate the roots of the cubic characteristic polynomial analytically. In the context of correspondence analysis, these roots referred to eigenvalues, which play an important role in assessing the quality of the correspondence plot. Considering the correspondence analysis on the I×J contingency table for I=4 and J=4,5,⋯, we obtained a cubic characteristic polynomial (since zero is one of its eigenvalues). Therefore, Cardano's formula allows us to obtain the eigenvalues directly without involving numerical processes, e.g., using singular value decomposition. We note several advantages of using Cardano's approach, such as (1) it produces the roots with the same result as singular value decomposition, as well more precise because without errors involving, (2) the algorithm is simpler and does not depend on initial guess, hence the computation time becomes shorter than numerical process, and (3) the manual calculation is easy because it uses a formula. The results show that the matrix operations on correspondence analysis can be replaced by a formula for determining eigenvalues and eigenvectors of the standard residual matrix directly. Some mathematical results are also presented.

## 1 Introduction

Cubic equations were widely known by ancient Babylonians, Greeks, Egyptians, Indians, and Chinese. In the 20^th^ century BC, the Babylonian cuneiform tablets have been found to solve cubic equations, but no evidence exists to confirm it (Cooke, 2008). Several Greeks, Egyptians, Indians mathematicians, given their effort to solve cubic equations graphically and by a formula. On the other hand, Chinese mathematicians developed numerical methods to solve them. Numerical methods represent encompasses techniques that not only approximate but also employ systematic strategies to reach the actual root (Chapra, 2012). From a linear algebra point of view, singular value decomposition (SVD) is the basic ideas and important contemporary applications that rely heavily on numerical ideas (Anton and Rorres, 2014).

In the early 15^th^ century, some Italian mathematicians found a formula for solving cubic equations, which is known as Cardano's formula. This formula discovered by Scipione del Ferro and Niccolò Tartaglia and published by Girolamo Cardano (1545) in his book Ars Magna (Cardano and Witmer, 1968; Irving, 2000). Cardano gave the general solution of reduced cubic polynomial-i.e a cubic equation with no second-degree term. He also provided methods to convert the general cubic equation to the reduced one (Gorroochurn, 2012). Cardano's contribution has led some researchers to attempt a systematic study of the calculus (*see* Osler, 2001; Fujii, 2003; Witula and Slota, 2010). The extension of Cardano's formula is also many widespread branches of mathematics, including statistics. For example, Cardano's formula used to study a non-Galois triple covering of algebraic surfaces (Tokunaga, 1991). Van Drop and Kotz (2002) had been estimated parameters of the triangular distribution by the method of moments using Cardano's formula. Liu et al. (2009) applied this formula to compute all the roots of a given polynomial within interval iteratively. Jiang et al. (2019) studied the contact process on trees with periodic degree sequences from solving the cubic equation by Cardano's formula. Later on, Lebensztayn and Utria (2019) investigated a system of branching random walk and established a new upper bound for the critical parameter of the model, which involves the Cardano's formula.

Extensively, the studies involving cubic equations are solved by the Cardano formula. Considering the CA of I×J contingency table for I=4 and J=4,5,..., this approach allows us to solve the cubic characteristic polynomial such that obtained the eigenvalue directly, without involving numerical processes, e.g., using SVD. By doing so, the matrix operation on CA can be replaced, and the result is more precise because without errors involving. Furthermore, Ginanjar et al. (2016) and Ginanjar (2017) introduced the simplification of correspondence analysis (SoCA) of 2×J and 3×J contingency table to obtain the principal coordinates. Some specific results presented firstly by proved that zero is a singular value of the standard residual matrix. This finding motivated us to extend this study on a 4×J contingency table. The theoretical aspect of Cardano's formula is briefly described in Section 2. In Section 3, we show how Cardano's approach led to resolving characteristic polynomials on correspondence analysis. Some mathematical results are also presented as lemmas. The case study put forward in Section 4. The summary is presented as a conclusion in Section 5.

## 2 Theory

### 2.1 Preliminary notation of correspondence analysis

Let $N=\left(n_{ij}\right)$ denote the I×J two-way contingency table that cross-tabulation n objects or individuals according to I row categories and J column categories. The (i,j)th cell entry of N is denoted by $n_{ij}$ for i=1,2,⋯,I, j=1,2,⋯,J, and $n=\sum_{i=1}^{I}\sum_{j=1}^{J}n_{ij}$. Define the ith row and jth column marginal frequency by $n_{i\cdot }=\sum_{j=1}^{J}n_{ij}$ and $n_{\cdot j}=\sum_{i=1}^{I}n_{ij}$, respectively. Denote $r={\left(n_{1\cdot },n_{2\cdot },\cdots ,n_{I\cdot }\right)}^{T}$ and $c={\left(n_{\cdot 1},n_{\cdot 2},\cdots ,n_{\cdot J}\right)}^{T}$to be vectors of the row and column marginal frequencies, respectively. Therefore, $D_{r}=\text{diag }\left(r\right)$ be the diagonal matrix of the row marginal frequencies, and $D_{c}=\text{diag }\left(c\right)$ be the diagonal matrix of the column marginal frequencies. The following contingency table N provides a summary of these notations (see Table 1).

**Table 1:** Notation of the I×J contingency table, consist of the joint frequency of items or individual that classified into the *i*th row and *j*th column category.

Column	Row						
	$C_{1}$	$C_{2}$	⋯	$C_{j}$	⋯	$C_{J}$	Sum
$R_{1}$	$n_{11}$	$n_{12}$	⋯	$n_{1j}$	⋯	$n_{1J}$	$n_{1\cdot }$
$R_{2}$	$n_{21}$	$n_{22}$	⋯	$n_{2j}$	⋯	$n_{2J}$	$n_{2\cdot }$
⋮	⋮	⋮		⋮		⋮	⋮
$R_{i}$	$n_{i1}$	$n_{i2}$	⋯	$n_{ij}$	⋯	$n_{iJ}$	$n_{i\cdot }$
⋮	⋮	⋮		⋮		⋮	⋮
$R_{I}$	$n_{I1}$	$n_{I2}$	⋯	$n_{Ij}$	⋯	$n_{IJ}$	$n_{I\cdot }$
Sum	$n_{\cdot 1}$	$n_{\cdot 2}$	⋯	$n_{\cdot j}$	⋯	$n_{\cdot J}$	$n=\sum_{i=1}^{I}\sum_{j=1}^{J}n_{ij}$

Let $P=n^{-1}N$ the correspondence matrix, and the (i,j)th element of P is the joint relative frequencies, denoted by $p_{ij}$ and $\sum_{i=1}^{I}\sum_{j=1}^{J}p_{ij}=1$. The ith row and jth column marginal proportions, respectively, define as $p_{i\cdot }=\sum_{j=1}^{J}p_{ij}$ and $p_{\cdot j}=\sum_{i=1}^{I}p_{ij}$. The vector of row and column marginal proportions denoted as $a={\left(p_{1\cdot },p_{2\cdot },\cdots ,p_{I\cdot }\right)}^{T}$ and $b={\left(p_{\cdot 1},p_{\cdot 2},\cdots ,p_{\cdot J}\right)}^{T}$, respectively. Thus, $D_{I}=\text{diag }\left(a\right)$ be the diagonal matrix of the row marginal proportions and $D_{J}=\text{diag }\left(b\right)$ be the diagonal matrix of the column marginal proportions. The ith row category by considering its relative frequency represented by *row profile*, $P_{r}=N^{T}D_{r}^{-1}$. While $P_{c}=ND_{c}^{-1}$ represents *column profile* for the jth column category. The columns of $P_{r}$ and $P_{c}$ matrices are vectors, each represented by a point, referred to as a *cloud of points*. The row clouds of points consist of J dimensions, and the column cloud of points consist of I dimensions (Beh and Lombardo, 2014).

Greenacre and Blasius (2006) represented the association in contingency table by the standardized residual matrix, $S=\left(s_{ij}\right)$ where $s_{ij}=\frac{n_{ij}-\frac{n_{i\cdot }n_{\cdot j}}{n}}{\sqrt{n_{i\cdot }n_{\cdot j}}}$ or in the matrix form

(1) $$S=D_{r}^{-\frac{1}{2}}\left(N-\frac{1}{n}rc^{\text{T}}\right)D_{c}^{-\frac{1}{2}}.$$

The sum of squared elements of S called the *total inertia*. It is referred to as the amount that quantifies the total variance in the contingency table and does not depend on the sample size (Greenacre, 2017).

### 2.2 Hypothesis of independence

CA uses the chi-squared statistic to measure the distance between points on the correspondence plot (Clausen, 1988). This distance also measures the associations among categorical variables. Let X and Ybe two categorical variables whereX consists of Icategories, and Yconsists of J categories. Let $P\left(X=i,Y=j\right)\approx p_{ij}$, $P\left(X=i\right)\approx p_{i\cdot }$, and, $P\left(Y=j\right)\approx p_{\cdot j}$. If X and Y are independent, then ${\hat{p}}_{ij}=p_{i\cdot }p_{\cdot j}$ for i=1,2,⋯,I and j=1,2,⋯,J. The independence hypothesis form of Pearson chi-squared test given by

(2) $$H_{0}:p_{ij}=p_{i\cdot }p_{\cdot j}\;\;\;\text{vs}.\;\;\;\;H_{1}:p_{ij}\neq p_{i\cdot }p_{\cdot j}.$$

Non-rejection of $H_{0}$ indicates that two categorical variables are independent. Conversely, the rejection of $H_{0}$ leads to the acceptance of $H_{1}$ and indicates that there is an association between two variables. Compute Pearson chi-squared statistic (Pearson, 1904).

(3) $$\chi^{2}=\sum_{i=1}^{I}\sum_{j=1}^{J}\frac{{\left(n_{ij}-\frac{n_{i\cdot }n_{\cdot j}}{n}\right)}^{2}}{\frac{n_{i\cdot }n_{\cdot j}}{n}}$$

to test the null hypothesis of independence. If $\chi^{2}>\chi_{\alpha }^{2}$ with ν=(I−1)(J−1) degree of freedom, then $H_{0}$ is rejected at the α-level of significance. Otherwise, fail to reject $H_{0}$ (Walpole et al., 2012).

### 2.3 Singular value decomposition (SVD)

Particularly, the SVD provides a straightforward mechanism of approximating an m×n matrix with another matrix of lower rank by least squared (Greenacre, 2017). In the context of CA, the association structure in the standardized residual matrix S is revealed using SVD;

(4) $$\begin{array}{c} S\\ \left(I\times J\right) \end{array}=\begin{array}{c} U\\ \left(I\times H\right) \end{array}\begin{array}{c} D_{\sigma }\\ \left(H\times H\right) \end{array}\begin{array}{c} V^{\text{T}}\\ \left(H\times J\right) \end{array},$$

where $D_{\sigma }$ is a diagonal matrix of singular values in descending order $\left(\sigma_{1}\geq \sigma_{2}\geq \cdots \geq \sigma_{H}\right)$ and H is rank of matrix S. The columns of U and V are orthonormal: $U^{\text{T}}U=I=V^{\text{T}}V$, called *left singular vectors* and *right singular vector*, respectively. The connection between the SVD and the eigen-decomposition shown below

(5a) $$SS^{\text{T}}=UD_{\sigma }V^{\text{T}}VD_{\sigma }U^{\text{T}}=UD_{\sigma }^{2}U^{\text{T}}=UD_{\lambda }U^{\text{T}},$$

(5b) $$S^{\text{T}}S=VD_{\sigma }U^{\text{T}}UD_{\sigma }V^{\text{T}}=VD_{\sigma }^{2}V^{\text{T}}=VD_{\lambda }V^{\text{T}}.$$

Thus, the squared singular values $\sigma^{2}$ in $D_{\sigma }^{2}$ correspond to the eigenvalues λ of $SS^{\text{T}}$ or $S^{\text{T}}S$. Similarly, the left singular vectors of S correspond to the eigenvectors of $SS^{\text{T}}$, and the right singular vectors correspond to the eigenvectors $S^{\text{T}}S$. With respect to CA, these eigenvalues are referred to as *principal inertias*, and their sum is equal to the total inertia, since

(6) $$\sum_{i=1}^{I}\sum_{j=1}^{J}s_{ij}^{2}=\text{trace}\left(SS^{\text{T}}\right)=\text{trace}\left(S^{\text{T}}S\right)=\text{trace}\left(D_{\sigma }^{2}\right)=\text{trace}\left(D_{\lambda }\right)$$

The SVD provides all the results that required to produce the CA plot. To obtained a symmetrical display, the principal row (R) and column coordinates (C) are used and calculated by

(7) $$R=D_{I}^{-\frac{1}{2}}UD_{\sigma }\;\;\text{and}\;\;C=D_{J}^{-\frac{1}{2}}VD_{\sigma }.$$

The columns of these matrices refer to the *principal axes* or *dimensions*. The mapping of the column vectors of R and C produces a plot called a *correspondence plot* that depicted the association of rows and columns in the contingency table. Two or more categories that are associated mapped close together and conversely. The category mapped close to the origin indicates that this category does not contribute to the association structure among variables and conversely. In particular, the first two columns of matrices R and C are the coordinates for a two-dimensional plot.

### 2.4 Cardano's formula

Considering SVD of S, the squared singular values $\sigma^{2}$ correspond to the eigenvalues λ of $SS^{\text{T}}$ or $S^{\text{T}}S$. These eigenvalues are the solutions of the equation $\det\left(\lambda I-SS^{\text{T}}\right)=0$ or $\det\left(\lambda I-S^{\text{T}}S\right)=0$. In the case of those equations result in a cubic characteristics polynomial, we use this polynomial to find all eigenvalues that correspondence to the squared singular value of S.

Suppose a cubic equation

(8) $$\lambda^{3}+b\lambda^{2}+c\lambda +d=0,$$

where b, c, and d are real numbers. By replacing $\lambda =\phi -\frac{b}{3}$, Eq. (8) can rewrite as

(9) $${\left(\phi -\frac{b}{3}\right)}^{3}+b{\left(\phi -\frac{b}{3}\right)}^{2}+c\left(\phi -\frac{b}{3}\right)+d=0.$$

Expand this out and cancel terms, then the $\varphi^{2}$ terms cancel, leading to a reduced cubic equation of form $\phi^{3}+p\phi +q=0$ where $p=c-\frac{b^{2}}{3}$ and $q=d-\frac{bc}{3}+\frac{2b^{3}}{27}$. To find a root of the reduced cubic polynomial $\varphi^{3}+p\varphi +q$, the renowned solution called *Cardano's formula* is available. The formula states that a solution is given by

(10) $$\lambda =\phi -\frac{b}{3}=-\frac{b}{3}+{\left(-\frac{q}{2}+{\left(\frac{p^{3}}{27}+\frac{q^{2}}{4}\right)}^{\frac{1}{2}}\right)}^{\frac{1}{3}}+{\left(-\frac{q}{2}-{\left(\frac{p^{3}}{27}+\frac{q^{2}}{4}\right)}^{\frac{1}{2}}\right)}^{\frac{1}{3}}$$

Use this solution to factor a reduced cubic equation and find the other two solutions by quadratic equation formula. Consider a cubic polynomial $\lambda^{3}+b\lambda^{2}+c\lambda +d$, suppose that $\lambda_{1},\lambda_{2},$ and $\lambda_{3}$ are its roots. Irving (2000) shows that the nature of the roots of a cubic polynomial is determined by the quantity $\text{Δ}={\left(\lambda_{1}-\lambda_{2}\right)}^{2}{\left(\lambda_{1}-\lambda_{3}\right)}^{2}{\left(\lambda_{2}-\lambda_{3}\right)}^{2}=-4p^{3}-27q^{2}.$ Based on this quantity, there are three natures of the cubic polynomial roots: 1) if Δ<0, it has one real root and two nonreal complex conjugate roots; 2) if Δ=0, it has a multiple roots and all its roots being real; and 3) if Δ>0, it has three distinct real roots.

However, finding the roots of a polynomial in R[λ] can be a hard matter, even if one knows that they exist. Some history records that no one was able to find a general procedure to determine the roots of any higher-degree polynomials. Over the two centuries of an attempt to find such a formula failed, speculation arose that perhaps no such formula existed. As proved by Niels Henrik Abel during the 1820s (Sørensen, 2010) that indeed, there could be no such formula. Later on, Evariste Galois during 1811–1832 (Irving, 2000) gave a proof of the more general fact that for every integer ngreater than four there cannot be a formula for the roots of a general degree-n polynomial in terms of the polynomial's coefficients and extraction of suitable mth roots. For this reason, we confined attention to generating the roots of a cubic characteristic polynomial by Cardano's approach in the context of the CA for I×J contingency, where I=4 and J=4,5,⋯.

Even though we cannot obtain the formulation to determine the roots of an arbitrary higher-degree polynomial in R[λ], the following theorem allows us to study factorization questions in R[λ].

Theorem 1 (Irving, 2000) *Let* ω(λ) *be a polynomial in* R[λ] *of positive degree* n*, then*

a. The polynomial ω(λ) factor in R[λ] as the product of polynomials of degree 1 or 2.
b. *The polynomial* ω(λ) *has n roots in* C *(counting multiplicity). In particular, there are nonnegative integer r and s satisfying* r+2s=n *such that* w(λ) *has r real roots and s pairs of nonreal conjugate complex numbers as roots.*
c. *The polynomial* ω(λ) *factor in* C[λ] *as the product of n degree-one polynomials.*

For more details, the other extension of Cardano's formula for higher-degree polynomial, *see* Witula and Slota (2010). They show a theoretical framework on the algebraic equation by rescaled Vieta-Lucas polynomials and Vieta-Fibonacci function to derive equations for generating the roots. However, the Cardano formula can still apply for general contingency tables without being limited in size. In the case of the I×J contingency table for I≥4, we use hierarchical clustering in the first step to reduce the categories (*see* Blasius and Greenacre, 1998; Renchers and Christensen, 2012; Greenacre, 2017). This stage aims to get four main categories so that the Cardano formula can be applied later.

## 3 Results and discussion

### *3.1* *Eigenvalues by Cardano*'*s formula*

Since the matrix product of the form $SS^{\text{T}}$ or $S^{\text{T}}S$ will play an important role in our study, we will begin with the basic properties of them. Ginanjar et al. (2014) defined that the element of $\dot{S}=SS^{\text{T}}$ is

(11) $$\dot{S}\;=\left({\dot{s}}_{ik}\right),\text{ with}{\dot{s}}_{ik}=\frac{1}{\sqrt{n_{i\cdot }n_{k\cdot }}}\left(\sum_{j=1}^{J}\frac{n_{ij}n_{kj}}{n_{\cdot j}}-\frac{n_{i\cdot }n_{k\cdot }}{n}\right)\text{for}\;\;\;\;i,k=1,2,\cdots ,I.$$

If I<J, then the size of the matrix $SS^{\text{T}}<S^{\text{T}}S$, thereby computing eigenvalues of $SS^{\text{T}}$, will be simplified. Since $SS^{\text{T}}$ is a real symmetric matrix, it has the following properties (Jacob, 1990): (1) $SS^{\text{T}}$ is positive semidefinite, then the eigenvalues are positive or zero with the number of positive eigenvalues equal to the rank of matrix (2) $SS^{\text{T}}$ is orthogonally diagonalizable, (3) The eigenvalues of $SS^{\text{T}}$ are real, and (4) $SS^{\text{T}}$ and $S^{\text{T}}S$ have the same rank, and their non zero eigenvalues are same. Additionally, Ginanjar (2016) proved that 0 is an eigenvalue of $SS^{\text{T}}$.

Theorem 2 (Ginanjar et al., 2016) *If the size of a matrix* N *is* I×J *and* $SS^{\text{T}}=\dot{S}$*, then 0 is eigenvalues of* $\dot{S}$.

Based on Theorem 1, the eigenvalues of 2×J and 3×J contingency table can be calculated, respectively, as follows.

**Lemma 1.** *Let* N *be the* 2×J *contingency table for* J=2,3,⋯*, and* $SS^{\text{T}}=\dot{S}$*, as in* Eq. (11)*. Suppose that* $b=-\sum_{i=1}^{2}{\dot{s}}_{ii}$*, and* $c={\dot{s}}_{11}{\dot{s}}_{22}-{\dot{s}}_{12}^{2}$, *then the eigenvalues that corresponding to* $\dot{S}$ *are* $\lambda_{1}={\dot{s}}_{11}+{\dot{s}}_{22}=-b$ *and* $\lambda_{2}=0$*.*

**Lemma 2.** *Let* N *be the* 3×J *contingency table for* J=3,4,⋯*, and* $SS^{\text{T}}=\dot{S}$*, as in* Eq. (11)*. Suppose that* $b=-\sum_{i=1}^{3}{\dot{s}}_{ii}$*,* $c=\sum_{\begin{array}{c} i=1\\ i\neq j \end{array}}^{2}\sum_{j=2}^{3}{\dot{s}}_{ii}{\dot{s}}_{jj}-\sum_{\begin{array}{c} i=1\\ i\neq j \end{array}}^{2}\sum_{j=2}^{3}{\dot{s}}_{ij}^{2}$, *and* $d=\sum_{\begin{array}{c} i=1\\ i\neq j\neq k \end{array}}^{3}\sum_{j=1}^{2}\sum_{k=2}^{3}{\dot{s}}_{ii}{\dot{s}}_{jk}^{2}-{\dot{s}}_{11}{\dot{s}}_{22}{\dot{s}}_{33}-2{\dot{s}}_{12}{\dot{s}}_{13}{\dot{s}}_{23}$, *then the eigenvalues that corresponding to* $\dot{S}$ *are* $\lambda_{1}=\frac{-b+\sqrt{b^{2}-4c}}{2}$*,* $\lambda_{2}=-b-\lambda_{1}$*, and* $\lambda_{3}=0$*.*

Lemma 1 and Lemma 2 are the other ways of presenting Lemma II.4 and Lemma III.1 by Ginanjar (2017). Proof of these Lemma can be studied in Ginanjar (2017). Extend to 4×J contingency table for J=4,5,⋯*,* the eigenvalues can be calculated directly by Cardano's approach, which is described in Lemma 3.

**Lemma 3.** *Let* N *be the* 4×J *contingency table for* J=4,5,⋯*, and* $SS^{\text{T}}=\dot{S}$*, as in* Eq. (11)*. Suppose that*

$$b=-\sum_{i=1}^{4}{\dot{s}}_{ii},$$

$$c=\sum_{\begin{array}{c} i=1\\ i\neq j \end{array}}^{3}\sum_{j=2}^{4}{\dot{s}}_{ii}{\dot{s}}_{jj}-\sum_{\begin{array}{c} i=1\\ i\neq j \end{array}}^{3}\sum_{j=2}^{4}{\dot{s}}_{ij}^{2},$$

$$d=\sum_{\begin{array}{c} i=1\\ i\neq j\neq k \end{array}}^{4}\sum_{j=1}^{3}\sum_{k=2}^{4}{\dot{s}}_{ii}{\dot{s}}_{jk}^{2}-\sum_{\begin{array}{c} i=1\\ i\neq j\neq k \end{array}}^{2}\sum_{j=2}^{3}\sum_{k=3}^{4}{\dot{s}}_{ii}{\dot{s}}_{jj}{\dot{s}}_{kk}-2\sum_{\begin{array}{c} i=1\\ i\neq j\neq k \end{array}}^{2}\sum_{j=2}^{3}\sum_{k=3}^{4}{\dot{s}}_{ij}{\dot{s}}_{ik}{\dot{s}}_{jk},$$

$$e={\dot{s}}_{11}{\dot{s}}_{22}{\dot{s}}_{33}{\dot{s}}_{44}+2\sum_{\begin{array}{c} i=1\\ i\neq j\neq k\neq l \end{array}}^{4}\sum_{j=1}^{4}\sum_{k=2}^{3}\sum_{l=3}^{4}{\dot{s}}_{ii}{\dot{s}}_{jk}{\dot{s}}_{jl}{\dot{s}}_{kl}-\sum_{\begin{array}{c} i=1\\ i\neq j\neq k\neq l \end{array}}^{3}\sum_{j=2}^{4}\sum_{k=1}^{3}\sum_{\ell =2}^{4}{\dot{s}}_{ii}{\dot{s}}_{jj}{\dot{s}}_{kl}^{2}-2\sum_{\begin{array}{c} i=2\\ i\neq j\neq k\neq l \end{array}}^{3}\sum_{j=3}^{4}\sum_{k=3}^{4}\sum_{l=2}^{3}{\dot{s}}_{1i}{\dot{s}}_{1j}{\dot{s}}_{2k}{\dot{s}}_{l4}+\sum_{\begin{array}{c} i=2\\ i\neq j\neq k\neq l \end{array}}^{4}\sum_{j=2}^{3}\sum_{k=3}^{4}{\dot{s}}_{1i}^{2}{\dot{s}}_{jk}^{2},\;$$

then the eigenvalues that corresponding to $\dot{S}$ are

$\lambda_{1}=-\frac{b}{3}+\ell ,$ $\lambda_{2}=-\frac{b}{3}-\frac{\ell -\sqrt{\ell^{2}-4\left(c-\frac{b}{3}+\ell^{2}\right)}}{2}$*,* $\lambda_{3}=-\frac{2b}{3}-\ell -\lambda_{2}$*, and* $\lambda_{4}=0,$ *where*

$$\ell ={\left(-\frac{\left(d-\frac{bc}{3}+\frac{2b^{3}}{27}\right)}{2}+{\left(\frac{{\left(c-\frac{b^{2}}{3}\right)}^{3}}{27}+\frac{{\left(d-\frac{bc}{3}+\frac{2b^{3}}{27}\right)}^{2}}{4}\right)}^{\frac{1}{2}}\right)}^{\frac{1}{3}}+{\left(-\frac{\left(d-\frac{bc}{3}+\frac{2b^{3}}{27}\right)}{2}-{\left(\frac{{\left(c-\frac{b^{2}}{3}\right)}^{3}}{27}+\frac{{\left(d-\frac{bc}{3}+\frac{2b^{3}}{27}\right)}^{2}}{4}\right)}^{\frac{1}{2}}\right)}^{\frac{1}{3}}$$

**Proof.** Consider to $\underset{\left(4\times J\right)}{S}$ for J=4,5,⋯*,* since $\dot{S}=SS^{\text{T}}$ is the 4×4 real symmetric matrix. The characteristic equation of $\dot{S}$ obtained from $\text{det}\left(\text{λ}I-\dot{S}\right)=0.$ Letb,c,d and e are the real numbers as given, then $\lambda^{4}+b\lambda^{3}+c\lambda^{2}+d\lambda +e=0$ is this is characteristic equation of $\dot{S}$. The fact in Theorem 2 implies that the characteristic equation of $\dot{S}$ takes a cubic equation form $\lambda^{3}+b\lambda^{2}+c\lambda +d=0.$ Set $\lambda =\phi -\frac{b}{3}$ and substitute to obtain a reduced cubic equation*.* By Cardano's formula, the solutions in Eq. (10) as follows

$$\ell ={\left(-\frac{\left(d-\frac{bc}{3}+\frac{2b^{3}}{27}\right)}{2}+{\left(\frac{{\left(c-\frac{b^{2}}{3}\right)}^{3}}{27}+\frac{{\left(d-\frac{bc}{3}+\frac{2b^{3}}{27}\right)}^{2}}{4}\right)}^{\frac{1}{2}}\right)}^{\frac{1}{3}}+{\left(-\frac{\left(d-\frac{bc}{3}+\frac{2b^{3}}{27}\right)}{2}-{\left(\frac{{\left(c-\frac{b^{2}}{3}\right)}^{3}}{27}+\frac{{\left(d-\frac{bc}{3}+\frac{2b^{3}}{27}\right)}^{2}}{4}\right)}^{\frac{1}{2}}\right)}^{\frac{1}{3}}.$$

Notice that by direct substitution, the reduced cubic equation has the solution ℓ. By using this solution to factor $\phi^{3}+p\phi +q$, find the other two solutions as follows

$\phi_{1}=\ell$, $\phi_{2}=\frac{-\ell -\sqrt{\ell^{2}-4\left(c-\frac{b^{2}}{3}+\ell^{2}\right)}}{2}$, and $\phi_{3}=\frac{-\ell +\sqrt{\ell^{2}-4\left(c-\frac{b^{2}}{3}+\ell^{2}\right)}}{2}.$

Since $\lambda =\phi -\frac{b}{3}$, $\lambda_{4}=0$, and $\sum_{h=1}^{4}\lambda_{h}=\text{trace}\left(\dot{S}\right)=-b$, such that

$\lambda_{1}=-\frac{b}{3}+\ell$*,* $\lambda_{2}=-\frac{b}{3}-\frac{\ell -\sqrt{\ell^{2}-4\left(c-\frac{b^{2}}{3}+\ell^{2}\right)}}{2}$*,* $\lambda_{3}=-\frac{2b}{3}-\ell -\lambda_{2}$*,* and of course $\lambda_{4}=0$*.*

### 3.2 Orthonormal eigenvectors

The following lemmas are intended to obtain the orthonormal eigenvectors corresponding to $\lambda_{h}$. The orthonormal eigenvectors are the column of the matrix U in Equation (5a). Ginanjar (2017) determined these vectors directly from the elements of the contingency table and state in Lemma 4 and Lemma 5. For simplify, write $\left({\text{λ}}_{h}-{\dot{s}}_{ii}\right)$ as ${\text{λ}}_{h,i}$.

**Lemma 4.** *Let* N *be the* 2×J *contingency table for* J=2,3,⋯*, and* $SS^{\text{T}}=\dot{S}$*, as in* Eq. (11)*. Let* $\lambda_{h}$ *is an h-th eigenvalue of* $\dot{S}$ *such that* ${\tilde{u}}_{1h}=-{\dot{s}}_{12}$ *and* ${\tilde{u}}_{2h}={\text{λ}}_{h,1}$*, then the columns of* U *are the orthonormal eigenvector of* $\dot{S}$*that corresponding to* $\lambda_{h}$*, that is* $U=\left(u_{ih}\right),$ *where* $u_{ih}=\frac{{\tilde{u}}_{ih}}{\sqrt{\sum_{i=1}^{2}{\tilde{u}}_{ih}}}.$

**Lemma 5.** *Let* N *be the* 3×J *contingency table, for* J=3,4,⋯*, and* $SS^{\text{T}}=\dot{S}$*, as in* Eq. (11)*. Let* $\lambda_{h}$ *is h-th eigenvalue of* $\dot{S}$ *such that* ${\tilde{u}}_{1h}={\dot{s}}_{13}{\text{λ}}_{h,2}+{\dot{s}}_{23}{\dot{s}}_{12}$*,* ${\tilde{u}}_{2h}={\dot{s}}_{23}{\text{λ}}_{h,1}+{\dot{s}}_{13}{\dot{s}}_{21}$*, and* ${\tilde{u}}_{3h}={\text{λ}}_{h,1}{\text{λ}}_{h,2}-{\dot{s}}_{12}^{2}$*, then the columns of* U *is the orthonormal eigenvector of* $\dot{S}$*that corresponding to* $\lambda_{h}$*, that is* $U=\left(u_{ih}\right),$ *where* $u_{ih}=\frac{{\tilde{u}}_{ih}}{\sqrt{\sum_{i=1}^{3}{\tilde{u}}_{ih}}}.$

Lemma 4 and Lemma 5 are the other representation of Lemma II.5 and Lemma III.2 by Ginanjar (2017). Certainly, we can find proof in Ginanjar (2017). Based on Lemma 5, rewrite ${\tilde{u}}_{ih}$ in the form

$${\tilde{u}}_{ih}=\{\begin{array}{l} {\dot{s}}_{i3}{\text{λ}}_{h,k}+{\dot{s}}_{k3}{\dot{s}}_{ik},fori\neq kandi,k=1,2.\\ {\text{λ}}_{h,1}{\text{λ}}_{h,2}-{\dot{s}}_{12}^{2},fori=3. \end{array}$$

The orthonormal eigenvectors of the 4×J contingency table determine as follows.

**Lemma 6.** *Let* N *be the* 4×J *contingency table for* J=4,5,⋯*, and* $SS^{\text{T}}=\dot{S}$*, as in* Eq. (11)*. Let* $\lambda_{h}$ *is the h-th eigenvalue of* $\dot{S}$ *such that*

$${\tilde{u}}_{ih}=\left\{ \;\begin{array}{c} {\dot{s}}_{ij}{\dot{s}}_{4j}{\text{λ}}_{h,k}+{\dot{s}}_{ik}{\dot{s}}_{4k}{\text{λ}}_{h,j}+{\dot{s}}_{i4}{\text{λ}}_{h,j}{\text{λ}}_{h,k}+{\dot{s}}_{jk}\left({\dot{s}}_{ij}{\dot{s}}_{4k}+{\dot{s}}_{ik}{\dot{s}}_{4j}-{\dot{s}}_{i4}{\dot{s}}_{jk}\right),\;i\neq j\neq k\;and\;i,j,k=1,2,3,\;\\ {\text{λ}}_{h,1}{\text{λ}}_{h,2}{\text{λ}}_{h,3}-{\dot{s}}_{12}^{2}{\text{λ}}_{h,3}-{\dot{s}}_{13}^{2}{\text{λ}}_{h,2}-{\dot{s}}_{23}^{2}{\text{λ}}_{h,1}-2{\dot{s}}_{12}{\dot{s}}_{13}{\dot{s}}_{23},\;i=4\;\;\;\;\;\;\;\;\;\;\;\;\;\;\;\;\;\;\;\;\;\;\;\;\;\;\;\;\;\;\;\;\;\;\;\;\;\;\;\;\;\;\;\;\;\;\;\;\;\;\;\;\;\;\;\;\;\;\;\;\;\;\;\;\;\;\;\; \end{array}\right.$$

Then the columns of U is the orthonormal eigenvector of $\dot{S}$that corresponding to $\lambda_{h}$, that is$U=\left(u_{ih}\right),$ where $u_{ih}=\frac{{\tilde{u}}_{ih}}{\sqrt{\sum_{i=1}^{4}{\tilde{u}}_{ih}}}.$

**Proof*.*** Suppose that $\vec{u_{h}}$ is the eigenvector *of* $\dot{S}$ that corresponding to $\lambda_{h}$, for h=1,2,⋯,H. As widely known, by definition of eigenvector (Anton and Rorres, 2014), $\left({\text{λ}}_{h}I-\dot{S}\right)\vec{u_{h}}=\vec{0}.$ By Gauss-Jordan elimination, since $\dot{S}$ is a real symmetric matrix can be obtained

$$\left(\begin{array}{ll} & \begin{array}{l} I_{3\times 3}\\ 0 \end{array} \end{array}\begin{array}{ll} & -{\tilde{u}}_{1h}/{\tilde{u}}_{4h}\\ & -{\tilde{u}}_{2h}/{\tilde{u}}_{4h}-{\tilde{u}}_{3h}/{\tilde{u}}_{4h}0 \end{array}\right)\left(\begin{array}{c} u_{1h}\\ \begin{array}{c} u_{2h}\\ u_{3h}\\ u_{4h} \end{array} \end{array}\right)=\left(\begin{array}{c} 0\\ \begin{array}{c} 0\\ 0\\ 0 \end{array} \end{array}\right),$$

where ${\tilde{u}}_{ih}$ as given, such that $u_{ih}-\frac{{\tilde{u}}_{ih}}{{\tilde{u}}_{4h}}u_{4h}=0\to u_{ih}=\frac{{\tilde{u}}_{ih}}{{\tilde{u}}_{4h}}u_{4h},i=1,2,3$. According to Lemma 3, suppose $u_{4h}=0$ then the eigenvector is

$$\vec{u_{h}}={\left(u_{1h}\;u_{2h}\;u_{3h}\;u_{4h}\right)}^{T}={\left(t\frac{{\tilde{u}}_{1h}}{{\tilde{u}}_{4h}}\;t\frac{{\tilde{u}}_{2h}}{{\tilde{u}}_{4h}}\;t\frac{{\tilde{u}}_{3h}}{{\tilde{u}}_{4h}}\;t\right)}^{T}.$$

Since each element $\vec{u_{h}}$ is devided by

$$\vec{u_{h}}=\sqrt{{\left(t\frac{{\tilde{u}}_{1h}}{{\tilde{u}}_{4h}}\right)}^{2}+{\left(t\frac{{\tilde{u}}_{2h}}{{\tilde{u}}_{4h}}\right)}^{2}+{\left(t\frac{{\tilde{u}}_{3h}}{{\tilde{u}}_{4h}}\right)}^{2}+t^{2}}=\frac{t}{{\tilde{u}}_{4h}}\sqrt{{\tilde{u}}_{1h}+{\tilde{u}}_{2h}+{\tilde{u}}_{3h}+{\tilde{u}}_{4h}},$$

the eigenvector *of* $\dot{S}$ that corresponding to $\lambda_{h}$ is

$$\vec{u_{h}}={\left(u_{1h}\;u_{2h}\;u_{3h}\;u_{4h}\right)}^{T}={\left({\tilde{u}}_{1h}\;{\tilde{u}}_{2h}\;{\tilde{u}}_{3h}\;{\tilde{u}}_{4h}\right)}^{T}\frac{1}{\sqrt{{\tilde{u}}_{1h}+{\tilde{u}}_{2h}+{\tilde{u}}_{3h}+{\tilde{u}}_{4h}}}.$$

In matrix form as $U=\left(u_{ih}\right),$ *where* $u_{ih}=\frac{{\tilde{u}}_{ih}}{\sqrt{\sum_{i=1}^{4}{\tilde{u}}_{ih}}}$

Consider to SVD, the unit vector $u_{ih}$ are *left singular vectors* of $\dot{S}$ (Horn and Johnson, 2013). The *right singular vectors* of $\dot{S}$ are determined, as stated in Lemma, that put forward.

**Lemma 7.** *Let* N *be the* 2×J *contingency table for* J=2,3,⋯*, and* $SS^{\text{T}}=\dot{S}$*, as in* Eq. (11)*. Let* $\lambda_{h}$ *is the h-th eigenvalue of* $\dot{S}$ *and* $u_{ih}$ *be the i-th left singular vectors corresponding to* $\lambda_{h}$*. Then the unit vector* $v_{ih}$ *are right singular vectors corresponding to* $\lambda_{h}$*, that is*

$$v_{jh}=\sum_{i=1}^{2}\frac{s_{ij}u_{ih}}{\sqrt{\lambda_{h}}},forh=1,\cdots ,H.$$

**Lemma 8.** *Let* N *be the* 3×J *contingency table for* J=3,4,⋯*, and* $SS^{\text{T}}=\dot{S}$*, as in* Eq. (11)*. Let* $\lambda_{h}$ *is the h-th eigenvalue of* $\dot{S}$ *and* $u_{ih}$ *be the i-th left singular vectors corresponding to* $\lambda_{h}$*. Then the unit vector* $v_{ih}$ *are right singular vectors corresponding to* $\lambda_{h}$*, that is*

$$v_{jh}=\sum_{i=1}^{3}\frac{s_{ij}u_{ih}}{\sqrt{\lambda_{h}}},forh=1,2,\cdots ,H.$$

As did before, Lemma 7 and Lemma 8 are other statements of Lemma II.5 and Lemma III.2 by Ginanjar (2017), the proof is also included. Now, let us extend to the 4×J contingency table.

**Lemma 9.** *Let* N *be the* 4×J *contingency table for* J=4,5,⋯*, and* $SS^{\text{T}}=\dot{S}$*, as in* Eq. (11)*. Let* $\lambda_{h}$ *is the h-th eigenvalue of* $\dot{S}$ *and* $u_{ih}$ *be the i-th left singular vectors corresponding to* $\lambda_{h}$*. Then the unit vector* $v_{ih}$ *are right singular vectors corresponding to* $\lambda_{h}$*, that is*

$$v_{kh}=\sum_{i=1}^{4}\frac{s_{ik}u_{ih}}{\sqrt{\lambda_{h}}},fork=1,2,\cdots ,J,andh=1,2,\cdots ,H.$$

**Proof.** Let $S=UDV^{\text{T}}$ be a *singular* value decomposition. Since U, V are unitary matrices and $D=\text{diag}\left(\vec{\sigma_{h}}\right)$, for h=1,2,⋯,H. Since $\sigma_{h}=\sqrt{\lambda_{h}}$ and $V^{\text{T}}=D^{-1}U^{\text{T}}S$, we have

$V^{\text{T}}=\left(v_{hj}\right)$**,** where $v_{hj}=s_{1j}\frac{u_{1h}}{\sqrt{\lambda_{h}}}+\cdots +s_{4j}\frac{u_{4h}}{\sqrt{\lambda_{h}}}=\sum_{i=1}^{4}\frac{s_{ij}u_{ih}}{\sqrt{\lambda_{h}}},\text{for }h=1,2,\cdots ,H$, then
$V=\left(v_{kh}\right)$**,** where $v_{kh}=\sum_{i=1}^{4}\frac{s_{ik}u_{ih}}{\sqrt{\lambda_{h}}},\text{for }k=1,2,\cdots ,J\text{ and }h=1,2,\cdots ,H$

### 3.3 Principal coordinates of row and column

By using previous lemmas, a formula will be found to determine the principal coordinates. Eq. (7) show that principal coordinates are a linear combination of eigenvectors of the association between the row and column categories. Lemma below shows that principal coordinates can be calculated directly from the elements of the standard residual matrix.

**Lemma 10.** *Let* N *be the* 4×J *contingency table for* J=4,5,⋯*, and* $SS^{\text{T}}=\dot{S}$*, as in* Eq. (11)*, then the row principal coordinates* (R) *is*

$$R=\left(r_{ih}\right),wherea_{ih}=\sqrt{\frac{\lambda_{h}}{p_{i\cdot }\sum_{i=1}^{4}{\tilde{u}}_{ih}}}{\tilde{u}}_{ih},$$

and column coordinates (C) is

$$C=\left(c_{jh}\right),whereb_{jh}=\sqrt{\frac{1}{p_{\cdot j}\sum_{i=1}^{4}{\tilde{u}}_{ih}}}\sum_{i=1}^{4}s_{ij}{\tilde{u}}_{ih},$$

*where* $\lambda_{h}$ *is the h-th eigenvalue of* $\dot{S}$*,* $p_{i\cdot }$ *is the* i*-th row marginal proportions,* $p_{\cdot j}$ *is the* j*-th column marginal proportions, and*

$${\tilde{u}}_{ih}=\left\{ \begin{array}{l} {\dot{s}}_{ij}{\dot{s}}_{4j}{\text{λ}}_{h,k}+{\dot{s}}_{ik}{\dot{s}}_{4k}{\text{λ}}_{h,j}+{\dot{s}}_{i4}{\text{λ}}_{h,j}{\text{λ}}_{h,k}+{\dot{s}}_{jk}\left({\dot{s}}_{ij}{\dot{s}}_{4k}+{\dot{s}}_{ik}{\dot{s}}_{4j}-{\dot{s}}_{i4}{\dot{s}}_{jk}\right),\;\;\;\;\;\;\;i\neq j\neq kandi,j,k=1,2,3,\\ {\text{λ}}_{h,1}{\text{λ}}_{h,2}{\text{λ}}_{h,3}-{\dot{s}}_{12}^{2}{\text{λ}}_{h,3}-{\dot{s}}_{13}^{2}{\text{λ}}_{h,2}-{\dot{s}}_{23}^{2}{\text{λ}}_{h,1}-2{\dot{s}}_{12}{\dot{s}}_{13}{\dot{s}}_{23},i=4 \end{array}\right.$$

**Proof.** According to Eq. (7), we have $R=\left(r_{ih}\right)$ where $r_{ih}=\sqrt{\frac{\lambda_{h}}{p_{i\cdot }}}u_{ih}$ and $C=\left(c_{jh}\right)$ where $c_{jh}=\sqrt{\frac{\lambda_{h}}{p_{\cdot j}}}v_{jh}$. Since $u_{ih}=\frac{{\tilde{u}}_{ih}}{\sqrt{\sum_{i=1}^{4}{\tilde{u}}_{ih}}}$ and ${\tilde{u}}_{ih}$ as given, by applying Lemma 6, it is easy to infer that the row principal coordinates is $R=\left(r_{ih}\right),$where$r_{ih}=\sqrt{\frac{\lambda_{h}}{p_{i\cdot }\sum_{i=1}^{4}{\tilde{u}}_{ih}}}{\tilde{u}}_{ih}.$

Similarly, since $v_{jh}=\sum_{i=1}^{4}\frac{s_{ij}u_{ih}}{\sqrt{\lambda_{h}}},$ for j=1,2,⋯,J and h=1,2,⋯,H, then the elements of C is $c_{jh}=\sqrt{\frac{1}{p_{\cdot j}\sum_{i=1}^{4}{\tilde{u}}_{ih}}}\sum_{i=1}^{4}s_{ij}{\tilde{u}}_{ih}$

## 4 Application in practical data analysis

An application to real-life data on the labor force is given in this section. The data obtained from the website of the Central Agency on Statistics Indonesia (BPS), www.bps.go.id. It is made up of 4 rows and 5 columns on 178,501,727 subjects and concerns the counts of different job status from five main islands in Indonesia. Each subject was classified into one of four statuses: “employed”, “unemployed”, “student”, and “others” (row of Table 2). These “main islands” classes have been cross-tabulated against “employment status” with five categories arranging from “Sumatera”, “Java”, “Kalimantan”, “Sulawesi to “Papua” (column of Table 2). An attempt will be made to use CA as a means to understand how the employment status associated with any islands. Furthermore, we modify the data to investigate the changes in the frequency of a cell and its effect on eigenvalues. Modifications were made by reducing the frequency of “employed-Java" by 19,000 subjects, then adding them to the frequencies of the other 19 cells, with as many as 10.000 subjects for each cell, as recorded along with the original data in Table 2.

**Table 2:** Cross-tabulation of the frequency of four employment status types across eight provinces in Indonesia at Agustus 2018, along with the modified data (in parentheses).

Employment status	Main islands					Sum
	Sumatera	Java	Kalimantan	Sulawesi	Papua	
Employed	26,436,332	**70,932,221**	7,646,839	8,907,164	2,092,906	116,015,46
	(26,446,332)	(70,742,221)	(7,656,839)	(8,917,164)	(2,102,906)	(115,865,462)
Unemployed	1,479,680	4,348,330	384,889	394,004	102,683	6,709,586
	(1,489,680)	(4,358,330)	(394,889)	(404,004)	(112,683)	(6,759,586)
Student	3,333,631	8,555,741	900,012	1,110,780	255,191	14,155,355
	(3,343,631)	(8,565,741)	(910,012)	(1,120,780)	(265,191)	(14,205,355)
Others	8,541,228	27,044,470	2,379,606	3,208,672	447,348	41,621,324
	(8,551,228)	(27,054,470)	(2,389,606)	(3,218,672)	(457,348)	(41,671,324)
Sum	39,790,871	110,880,762	11,311,346	13,620,620	2,898,128	**178,501,727**
	(39,830,871)	(110,720,762)	(11,351,346)	(13,660,620)	(2,938,128)	

Cells in bold are used as a central to obtain modified data (in parentheses), by reducing the frequency of the cell by 19,000 subjects, then adding them to the frequencies of the other 19 cells, with as many as 10,000 subjects for each cell.

Consider the original data in Table 2, if there were no difference between the employment status as far as main islands is concerned, we would expect that the observed frequencies of rows (or columns) is more or less the same as their expected frequencies, and would differ from it only because of random sampling fluctuations. Assuming no difference means that the employment status and main islands are independent. For example, under the independence assumption, the expected frequencies for “employment” to be [25,861,689.7472,065,873.247,351,699.368.852,589.541,883,610.12], but in reality the observed to be [26,436,33270,932,2217,646,8398,907,1642,092,906]. It shows that the expected values are different from their corresponding observed values. Chi-squared statistics computed to measures how these differences are large enough to contradict the assumed hypothesis of independence. According to Eq. (3), the Pearson chi-squared statistics for both data are respectively $\chi_{\text{original}}^{2}=341,605.13$ and $\chi_{\text{modified}}^{2}=337,437.910$, while the critical value $\chi_{\alpha }^{2}$ is 21.03, which at 12 degrees of freedom. Since $\chi_{\text{original}}^{2}>\chi_{\alpha }^{2}$, and $\chi_{\text{modified}}^{2}>\chi_{\alpha }^{2}$, $H_{0}$ should be rejected. Thus, there is evidence suggesting that there is a statistically significant association between employment status and main islands for both the data.

The total inertia in the contingency table of the original data is0.001914, whereas if we perform the modified data, the total inertia turns out to be 0.001890. There is a loss of inertia when the frequency of a cell change. Referring to Eq. (6), the decrease in total inertia is due to the reduction in the total sum of the eigenvalues of $\dot{S}$. This shows that there is a difference in the eigenvalues since the data has been modified as exposes in Table 3.

**Table 3:** Comparison of finding roots analytically by Cardano's formula in Lemma 3 (since $\lambda_{4}=0)$ and numerically using SVD by bisection method for original and modified data.

Data		Roots	Initial guess; Iteration number; Error	Maximum computational time (sec)
Original	Cardano's formula	$\lambda_{1}=0.0017049968$	−	0.0011820793
		$\lambda_{2}=0.0001835046$	−	
		$\lambda_{3}=0.0000252343$	−	
	SVD	$\lambda_{1}=0.0017049968$	[0.001100;0.002000];$27;7.82822\cdot 10^{-21}$	0.0040500164
		$\lambda_{2}=0.0001835047$	[0.000110;0.000200];$17;4.36257\cdot 10^{-21}$	
		$\lambda_{3}=0.0000252344$	[0.000019;0.000027];$9;0.61893\cdot 10^{-21}$	
Modified	Cardano's formula	$\lambda_{1}=0.0016972128$	−	0.0010056496
		$\lambda_{2}=0.0001741585$	−	
		$\lambda_{3}=0.0000190189$	−	
	SVD	$\lambda_{1}=0.0016972128$	[0.001100;0.002000];$27;7.93289\cdot 10^{-21}$	0.0070011616
		$\lambda_{2}=0.0001741584$	[0.000110;0.000200];$18;2.34520\cdot 10^{-21}$	
		$\lambda_{3}=0.0000190189$	[0.000019;0.000027];$11;3.22775\cdot 10^{-21}$	

The difference in eigenvalues is not visible since the frequency of “employed-Java" decreased by 0.11% from the total frequency, while other cells have increased about 0.01%. For the other comparison, we tried the numeric process using SVD through the bisection method. Considering the number of iterations, error involvement, computational time, and algorithm, it appears that the Cardano's approach that we propose has advantages over the numerical approach, such as (1) it produces the roots with the same result as SVD, as well more precise because without errors involving, (2) the algorithm is simpler and does not depend on initial guess, hence the computation time becomes shorter than numerical process, and (3) the manual calculation is easy because it uses a formula.

**Figure 1:**
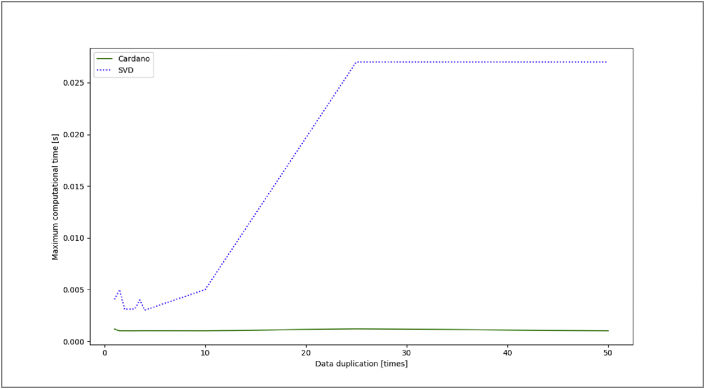
The maximum computational time for finding roots using Cardano's formula and SVD. The Cardano's formula produces a linear curve that indicates the stability in computational time, while SVD tends to be increased.

However, it should be noted that the number of iterations depends on the initial guess given. If the initial guess given is close to its analytic root, the less iteration is needed, and contrarily. While computing time also depends on the processor speed used when running the program (we used Python 3.8 by MacBook Air 13 Processor 1.6 GHz dual-core Intel Core i5, Turbo Boost up to 3.6 GHz with 4MB L3 cache). Furthermore, we simulate the original data by some duplication to investigate maximum computational time using Cardano's formula and SVD. The results show that Cardano's formula is faster than SVD, in terms of finding roots. This formula tends to be more stable when applied to big data (see Figure 1).

**Figure 2:**
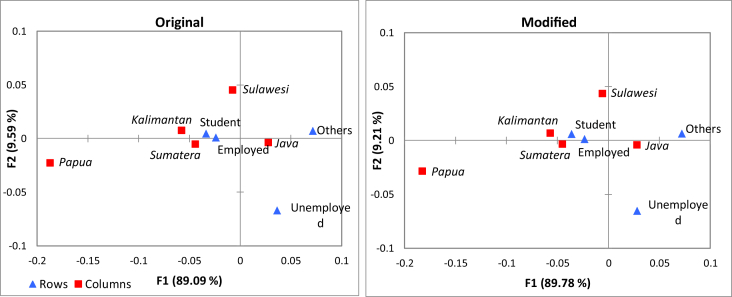
Two-dimensional plot of principal coordinates of the employment status data in Table 2. The points configuration on both plots is similar since the changes of frequencies for each cell are the same, and the difference of their eigenvalues is not noticeable.

By applying Lemma 6, 9, and 10 sequentially, the correspondence plot is displayed in Figure 2. These plots explain 98.68% and 98.99% of the inertia, respectively, that naturally depicts the association between employment status and main islands. It appears that row coordinate of “unemployed” and column coordinate of “Papua” are relatively far from the origin (0.0). Thus, the most important contributors are the unemployed status and Papua island. It means if the categories of “unemployed” or “Papua” are omitted, then the different results will be obtained.

Moreover, the position of “Kalimantan” and “Sumatera” are close to one another in the correspondence plot. Likewise, the position of “student” and “employed” are close together. These results lead to the conclusion that the profiles of Kalimantan and Sumatera island are similarly based on employment status.

## 5 Conclusion

The objective of this paper has been propose the Cardano's approach as the alternative solution to generate the roots of the cubic characteristic polynomial analytically. This approach allows one to obtain the eigenvalue directly, without involving numerical processes, e.g., using singular value decomposition (SVD). The main idea of this approach is to transform the cubic polynomial characteristic into the reduced cubic polynomial form, and its renowned formula called *Cardano's formula*. This formula is adequately sophisticated to find the roots of the cubic characteristic polynomial, namely the eigenvalues.

The computational algorithm to obtain the row and column principal coordinates are presented as lemmas. Consider to I×J contingency table for I=4 and J=4,5,⋯*,* the eigenvalues can be calculated directly by Cardano's formula as states in Lemma 3. The left and right singular vectors of residual standard matrix S correspond to the eigenvectors of $SS^{\text{T}}$ are given by Lemma 6 and Lemma 9, respectively. Finally, the row and column principal coordinates can be calculated directly from the elements of S by Lemma 10. The results show that the matrix operations on CA can be simplified by determining eigenvalue and eigenvector of the standard residual matrix analytically.

In case study to practical data analysis, by considering the number of iterations, error involvement, computational time, and algorithm, it appears that the Cardano's approach that we propose has advantages over the numerical approach. We note several advantages of using Cardano's approach, such as (1) it produces the roots with the same result as SVD, as well more precise because without errors involving, (2) the algorithm is simpler and does not depend on initial guess, hence the computation time becomes shorter than numerical process, and (3) the manual calculation is easy because it uses a formula. In the case of the I×J contingency table for I≥4, the Cardano formula can still apply for general contingency tables without being limited in size. We use hierarchical clustering in the first step to reduce the categories. This stage aims to get four main categories so that the Cardano formula can be applied later.

## Declarations

### Author contribution statement

K. E. Lestari: Conceived and designed the experiments; Performed the experiments; Analyzed and interpreted the data; Wrote the paper.

U. S. Pasaribu: Conceived and designed the experiments; Performed the experiments; Analyzed and interpreted the data; Contributed reagents, materials, analysis tools or data; Wrote the paper.

S. W. Indratno: Analyzed and interpreted the data; Contributed reagents, materials, analysis tools or data; Wrote the paper.

H. Garminia: Contributed reagents, materials, analysis tools or data; Wrote the paper.

### Funding statement

This research was supported by Lembaga Pengelola Dana Pendidikan (LPDP) from Ministry of Finance Indonesia, Indonesia through the scheme of BUDI-DN [grant number 20161141021036].

### Competing interest statement

The authors declare no conflict of interest.

### Additional information

No additional information is available for this paper.
